# Distinct overlapping functions for Prickle1 and Prickle2 in the polarization of the airway epithelium

**DOI:** 10.3389/fcell.2022.976182

**Published:** 2022-09-13

**Authors:** Koshi Kunimoto, Alexis T. Weiner, Jeffrey D. Axelrod, Eszter K. Vladar

**Affiliations:** ^1^ Department of Pathology, Stanford University School of Medicine, Stanford, CA, United States; ^2^ Department of Medicine, Division of Pulmonary Sciences and Critical Care Medicine, University of Colorado School of Medicine, Aurora, CO, United States

**Keywords:** cilia, planar cell polarity (PCP), prickle, airway, mouse

## Abstract

Planar cell polarity (PCP) signaling polarizes cells within the plane of an epithelium. In the airways, planar cell polarity signaling orients the directional beating of motile cilia required for effective mucociliary clearance. The planar cell polarity signaling mechanism is best understood from work in *Drosophila*, where it has been shown to both coordinate the axis of polarity between cells and to direct the morphological manifestations of polarization within cells. The ‘core’ planar cell polarity signaling mechanism comprises two protein complexes that segregate to opposite sides of each cell and interact with the opposite complex in neighboring cells. Proper subcellular localization of core planar cell polarity proteins correlates with, and is almost certainly responsible for, their ability to direct polarization. This mechanism is highly conserved from *Drosophila* to vertebrates, though for most of the core genes, mammals have multiple paralogs whereas *Drosophila* has only one. In the mouse airway epithelium, the core protein Prickle2 segregates asymmetrically, as is characteristic for core proteins, but is only present in multiciliated cells and is absent from other cell types. Furthermore, *Prickle2* mutant mice show only modest ciliary polarity defects. These observations suggest that other Prickle paralogs might contribute to polarization. Here, we show that Prickle1 segregates asymmetrically in multiciliated and nonciliated airway epithelial cell types, that compared to *Prickle2*, *Prickle1* has different spatial and temporal expression dynamics and a stronger ciliary polarity phenotype, and that *Prickle1* and *Prickle2* mutants genetically interact. We propose distinct and partially overlapping functions for the Prickle paralogs in polarization of the airway epithelium.

## Introduction

The planar cell polarity (PCP) pathway controls directional cell behaviors such as oriented division, directed migration, and polarized cell morphologies (Vladar et al., 2009). Its most well-known function is the polarization of epithelial cells and cellular structures along the plane of an epithelium, orthogonal to the apico-basal axis. PCP remains best characterized in *Drosophila*, where it controls the polarity of cuticular hairs and bristles. In vertebrates, it regulates the orientation of hair follicles and auditory hair cells, and notably the alignment of motile cilia for anatomically directed mucociliary clearance along the distal to proximal (D-P, lung to oral) axis of the airways (Wang and Nathans, 2007; Simons and Mlodzik, 2008; Vladar et al., 2012).

Underlying morphological polarization is the PCP signaling mechanism, through which upstream global directional cues instruct a core module that coordinates the polarity of neighboring cells and downstream intracellular effectors that polarize cellular structures via the cytoskeleton (Carvajal-Gonzalez and Mlodzik, 2014; Harrison et al., 2020). Best understood are the molecular mechanisms of the fly core module that relies on cell-cell communication to polarize neighboring cells with each other along the tissue axis (Harrison et al., 2020). At the heart of the mechanism are two protein complexes that segregate into opposing, asymmetrically localized membrane domains (“crescents”) at the proximal and distal apical cell junctions. The Vang/Pk crescent comprises Van Gogh (Vang; Vangl in mammals), Prickle (Pk) and Flamingo (Celsr in mammals), and the Fz/Dsh crescent contains Frizzled (Fz), Dishevelled (Dsh; Dvl in mammals) and Flamingo. Vang/Pk and Fz/Dsh complexes are initially randomly distributed at apical junctions, where intra- and intercellular interactions promote the formation of heterotypic Vang/Pk - Fz/Dsh complexes across adjacent membranes of neighboring cells. A biasing input and mutual exclusion of oppositely oriented complexes results in the coordinated tissue-wide segregation of Vang/Pk complexes to one side and Fz/Dsh complexes to the other side of cells.

The core mechanism shows remarkable evolutionary conservation in the vertebrate skin, inner ear and the multiciliated epithelia of the oviduct, ependyma and respiratory tract (Vladar et al., 2009; Butler and Wallingford, 2017). Core PCP protein homologs display crescent localization and mediate morphological changes by regulating cytoskeletal dynamics along the polarity axis (Yin et al., 2008; Luga et al., 2012; Kaucka et al., 2015). However, whereas flies have single genes and isoforms for most core components, the presence of multiple, often numerous, vertebrate PCP protein paralogs has hindered efforts to delineate detailed mechanisms. In the mammalian airways, the two Vangl homologs, Vangl1 and Vangl2 cooperate to polarize motile ciliary orientation (Vladar et al., 2012). Overlapping, but distinct contributions are indicated by identical patterns of expression, but more severe polarity phenotypes in Vangl1 compared to Vangl2 mutant mice.

The mouse airway epithelium has one of the best characterized vertebrate PCP mechanisms to date, where it controls the proximal orientation of motile cilia within multiciliated cells (Vladar et al., 2012; Vladar et al., 2016). Multiciliated cells each contain 200–300 cilia, whose directional movement mediates the mucociliary clearance of the lung, a fundamental host defense mechanism (Boutin and Kodjabachian, 2019). We showed that during mouse development core PCP proteins segregate into asymmetric membrane domains to form a Fz/Dvl crescent at the proximal (oral) and a Vangl/Pk crescent at the distal (lung) side of cells (Vladar et al., 2012). Cilia are physically oriented (and thus will beat) towards the proximal side via PCP-dependent microtubules that originate at the base of cilia and are captured by their plus ends at the Fz/Dvl crescent. In airways mutant for core components (ex. Vangl1), robust crescents fail to form, and cilia are misaligned. *In vitro* cultured PCP mutant primary airway epithelial layers also show diminished barrier capacity and regeneration, although mechanisms are not yet known (Vladar et al., 2016). In addition to mispolarized airway epithelia, PCP mutant mice also display airway axis elongation and distal lung morphogenesis defects (Li et al., 2002; Yates et al., 2010).

Prickle proteins play fundamental roles in the core PCP mechanism in flies (Harrison et al., 2020). Pk is a binding partner of Vang, and Vang is required for Pk membrane localization in flies and vertebrates (Bastock et al., 2003; Nagaoka et al., 2019). Studies suggest that Pk controls microtubule cytoskeleton organization in planar polarized cells, but knowledge of specific mechanisms, especially in vertebrate cells, is lacking (Shimada et al., 2001; Harumoto et al., 2010; Tao et al., 2011; Ehaideb et al., 2014; Matis et al., 2014; Olofsson et al., 2014). Our understanding of the vertebrate Pk family is limited. It is comprised of four (Pk1-4) paralogs, which each contain a Prickle-Espinas-Testin (PET) domain near the N-terminus, followed by three LIN110-Isl1-MEC3 (LIM) domains in Pk1, Pk2 and Pk3 and two in Pk4, and a C-terminal CAAX motif (Schenkelaars et al., 2016). Pk1-3 show crescent localization in various vertebrate tissues (Deans et al., 2007; Antic et al., 2010; Vladar et al., 2012). We demonstrated that Pk1-4 all are able to form crescents in primary cultured mouse airway epithelial cells (Vladar et al., 2016). Only Pk1 and Pk2 mutant mice have been described so far (Tao et al., 2009; Tao et al., 2011; Minegishi et al., 2017). Mutant phenotypes are highly sensitive to genetic background, with early embryonic lethality due to polarity defects in restrictive backgrounds. In one background, homozygous null mutation of Pk1 is embryonic lethal, but heterozygotes and homozygotes with missense mutations have been used to show that Pk1 is required for skeletal, inner ear and cardiac development (Gibbs et al., 2016). Pk2 was also implicated in controlling PCP in multiple developmental systems: airways, ependyma, neural tube, inner ear, renal tubules, and the embryonic node (Deans et al., 2007; Tao et al., 2012; Vladar et al., 2016). In a more permissive genetic background, Pk1 and Pk2 mutations cause seizures in mice, flies, and humans (Tao et al., 2011). Pk1 and Pk2 have been linked to autism with underlying defects in neuronal structure (Paemka et al., 2013; Sowers et al., 2013; Todd and Bassuk, 2018). Pk3 was shown to be required for the polarized placement of solitary cilia in the *Xenopus* nodal epithelium and the formation of multiciliated cells in the embryonic frog skin (Ossipova et al., 2015; Chu et al., 2016). Pk4 function has not been studied.

We previously showed that Pk2 is required for motile ciliary orientation (Vladar et al., 2016). However, Pk2 mice display a relatively mild ciliary orientation phenotype, and intriguingly, Pk2 is only expressed by multiciliated cells (Vladar et al., 2012). This raised the possibility that other Pk proteins contribute to core PCP regulation in the airway epithelium. Here, we show that all four Pk paralogs are expressed in the airway epithelium and display strikingly different temporal and cell-type dependent patterns of expression. They likely all act as part of the core PCP machinery, as they were all able to form Vangl1-dependent crescents in airway epithelial cells. Using knockout mice in a permissive, mixed genetic background, we show that Pk1 and Pk2 coordinately regulate ciliary planar polarization with quantitatively and qualitatively distinct roles and entirely distinct patterns of expression. The more severe ciliary phenotypes in Pk1 mutants suggest that it plays a more critical role, but genetic interaction displayed by double mutants clearly indicate that both Pks contribute to airway epithelial PCP. These studies lay the groundwork for delineation of specific functions of the Pk family of core proteins, and a more complete understanding of mechanisms driving ciliary polarity.

## Materials and methods

### Mice

Pk1 and Pk2 mutant mice used in the adult analyses were obtained from N. Ueno (Tao et al., 2009; Tao et al., 2011). A portion of exon 1 is deleted in the Pk1 mutants. Exons 4-6 are deleted in the Pk2 mutants. In the CBA background, mice are lethal at an early stage (Tao et al., 2009; Tao et al., 2012), whereas 8 times (complete) backcross into C57BL/6 resulted in good viability and the mice displayed an epilepsy and ataxia phenotype (Tao et al., 2011). Pk1 and Pk2 mutant mice used in the perinatal studies were obtained from K. Minegishi and H. Hamada (Minegishi et al., 2017). Exon 6 is deleted in both the Pk1 and Pk2 mutants, which results in the loss of the C-terminal region. Mice were maintained in a C57BL/6 x CBA mixed background.

### Primary mouse tracheal epithelial cell (MTEC) culture

Cell culture was carried out as previously described (Vladar and Brody, 2013). Briefly, tracheas were isolated and incubated overnight in Pronase (Sigma) solution to isolate epithelial cells. Cells were seeded onto Collagen I-coated 24-well size Transwell membranes (Corning) and cultured submerged in proliferation medium for approximately 5 days. Air-liquid interface (ALI) was created by adding differentiation medium to only the bottom compartment of the Transwell culture plate. MTECs are considered mature at 14 days after ALI creation.

### Lentiviral constructs and gene transfer

Lentiviral vectors containing GFP-tagged Prickle1-4 open reading frames have been previously described (Vladar et al., 2016). Lentivirus was prepared in the 293T/17 cell line (ATCC) using the psPAX2 and pMD2.G helper plasmids (Addgene) according to published methods. MTECs were infected on day three of culture after EGTA treatment to temporarily disrupt epithelial junctions followed by spin infection (Vladar and Brody, 2013).

### Immunofluorescence

MTEC cultures and mouse tracheas were fixed at −20°C in ice cold methanol for 10 min. For wholemount labeling, tracheas were opened longitudinally and pinned luminal side up onto Sylgard-184 elastomer (Ellsworth Adhesives) slabs. Samples were blocked in 10% normal horse serum and 0.1% Triton X-100 in PBS and incubated with primary antibodies for 1–2 h, then with Alexa dye conjugated secondary antibodies (Thermo Fisher) for 30 min at room temperature. Samples were mounted in Mowiol mounting medium containing 2% N-propyl gallate (Sigma). Specimens were imaged with a Leica SP8 confocal microscope. For antibodies, see Supplementary Table S1. We previously described the generation and testing of the Pk1 and Pk2 antibodies (Deans et al., 2007; Bassuk et al., 2008).

### Gene expression analysis

cDNA was prepared from MTECs using standard methods. cDNA from multiciliated (EGFP+) and other nonciliated (EGFP) cells was obtained by FACS cells from *Foxj1/EGFP* MTECs or adult tracheas as previously described (Ostrowski et al., 2003; Vladar and Stearns, 2007). qPCR was performed in triplicate with Power SYBR Green Master Mix (Thermo Fisher) in a StepOnePlus Real-Time PCR System (Thermo Fisher), and gene expression was evaluated using the ΔΔCt method. For primer sequences, see Supplementary Table S2.

### Transmission electron microscopy (TEM)

Tissue preparation and image acquisition were as previously described (Vladar et al., 2012; Vladar et al., 2015). Proximal airway direction was tracked throughout the procedure. Tracheas were fixed in 2% glutaraldehyde, 4% paraformaldehyde in 0.1 M NaCacodylate buffer, pH 7.4 at 4°C overnight. Samples were osmicated, stained with uranyl acetate, then dehydrated with a graded ethanol series and infiltrated with EMbed-812 (Electron Microscopy Sciences). 80–100 nm sections were mounted onto copper grids and analyzed with a JEOL JEM-1400 microscope using a Gatan Orius Camera.

### Quantitation of ciliary orientation

Basal body orientation in perinatal and adult mice was evaluated in TEM images showing the basal body layer in cross-section in multiciliated cells in the trachea. The proximal direction was set at 0°. Basal body orientation was evaluated by drawing a line representing the oral-lung axis and fitting a ±15° angle in the proximal direction using the angle tool in the Fiji software (NIH). Basal feet oriented within these 30° were scored as having correct polarity and basal feet that were oriented outside of the 30° were scored as having incorrect polarity. This binary score was then summed for each category within each genotype. Graphpad Prism software was used to graph data. For the perinatal tissues with low sample numbers, a Fisher’s exact test with two-tailed *t*-test was performed to compare groups. For the adult tissues with higher sample numbers, a simple one-way ANOVA test was used with Dunnett’s post hoc test for multiple hypothesis testing.

## Results

### Temporal and cell type specific expression of Pk family homologs in the airway epithelium

**FIGURE 1:**
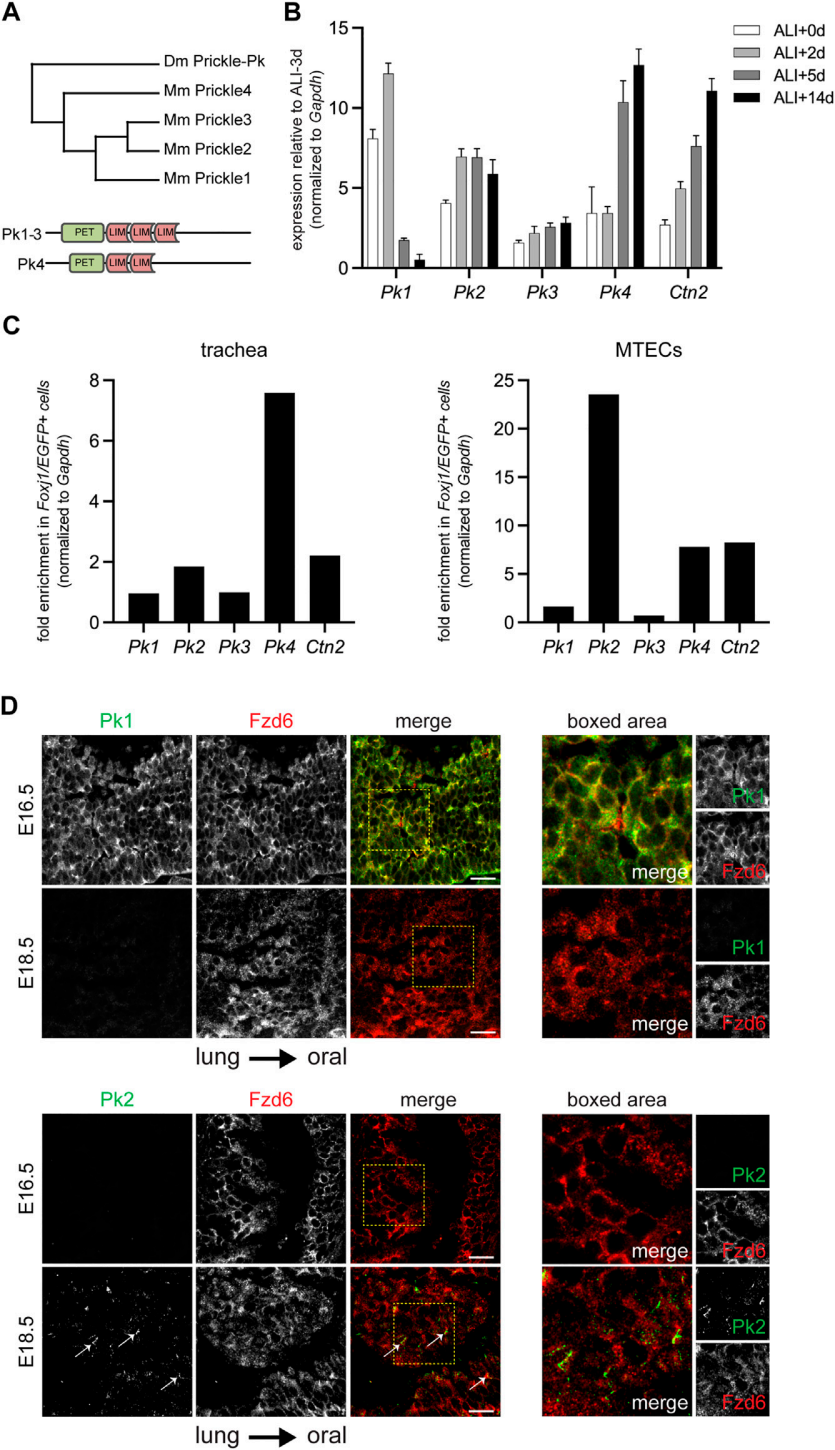
Temporal and cell type specific expression of Pk family members. **(A)**. Phylogram depicting the mouse and *Drosophila* Pk proteins with schematic of domain structures of mouse Pk1-4. **(B)**. qRT-PCR of mouse *Pk1-4* over a timecourse MTEC culture spanning early epithelialization (ALI+0-2 days), ciliogenesis (ALI+5 days), and maturity (ALI+14 days). Graph shows the average ± SE of n = 4 timecourses. The expression of *Centrin2* (*Ctn2*), a component of ciliary basal bodies is used to monitor ciliogenesis kinetics. **(C)**. qRT-PCR of mouse *Pk1-4* expression in multiciliated (*Foxj1/EGFP* positive) cells vs. other epithelial cells in the adult trachea (left) and in ALI+14 days mature MTECs (right). **(D)**. Maximum projections of confocal image stacks of wholemount immunolabeled E16.5 and E18.5 mouse embryonic tracheal lumens with Pk1 (left) or Pk2 (right) antibodies (green) and Fzd6 antibody (red). Arrows show emerging Pk2 crescents at E18.5. Boxed areas shown to the right. Scale bar, 20 μm. Note, due to tissue curvature, it was not always possible to mount the samples sufficiently level to capture the entire tissue layer in a single image stack.

Prior analysis of the contribution of Pk2 to PCP in the airway epithelium suggested a limited role in establishing or maintaining polarity. Pk2 is expressed in crescents that arise late during differentiation, well after other core PCP proteins are strongly polarized, and its expression is limited to multiciliated cells (Vladar et al., 2012). Furthermore, *Pk2* ^*−/−*^ mutants display only a mild ciliary polarity disruption phenotype (Vladar et al., 2016). We therefore explored the possibility that other Pk paralogs might participate in PCP signaling in this tissue. Pk2 is one of four structurally related paralogs (Pk1-4) in mice. Pk1, Pk2 and Pk3 are more closely related, followed by Pk4 (Figure 1A and Supplementary Figure S1A).

Because embryonic airway tissue is limited and cell turnover in the adult airway epithelium is asynchronous, we leveraged the roughly synchronous differentiation of primary mouse tracheal epithelial cell (MTEC) cultures to study gene expression timing and cell type specificity for the four Pk paralogs (Supplementary Figure S2A). qRT-PCR profiles show relative expression of *Pk1-4* mRNAs through MTEC differentiation (Figure 1B and Supplementary figure S2B). *Pk1* expression is highest early in differentiation, then declines rapidly, whereas *Pk4* is initially low, then increases through differentiation. *Pk2* and *Pk3* both increase modestly during the differentiation timecourse. Note, however, that previous protein expression analyses showed that Pk2 protein does not localize to the apico-lateral membrane until after basal body replication in multiciliated cells (Vladar et al., 2012), suggesting either that Pk2 protein is present but not membrane localized or is not translated until this stage. We then tested for multiciliated cell-enriched expression by sorting EGFP-labeled multiciliated cells from other epithelial cells derived from mature MTEC cultures and from adult tracheas from *Foxj1-EGFP* mice (Ostrowski et al., 2003; Vladar and Stearns, 2007). These analyses show that *Pk1* and *Pk3* are expressed at similar levels in multiciliated cells and in other epithelial cells, whereas *Pk2* (as shown previously) and *Pk4* are substantially enriched in multiciliated cells (Figure 1C and Supplementary Figure S2C).

Antibody labeling previously showed that Pk2 is expressed on the distal airway (lung) side of multiciliated cells (Vladar et al., 2012). Consistent with early pan-epithelial expression, wholemount Pk1 antibody labeling of the tracheal lumen detects weak junctional localization in all cells in very early tracheas (E16.5), when little or no Pk2 is detected, but no Pk1 signal is present in slightly more mature tracheas (E18.5) when Pk2 is beginning to be detected (Figure 1D).

### Prickle family asymmetric membrane localization depends on Vangl1

**FIGURE 2:**
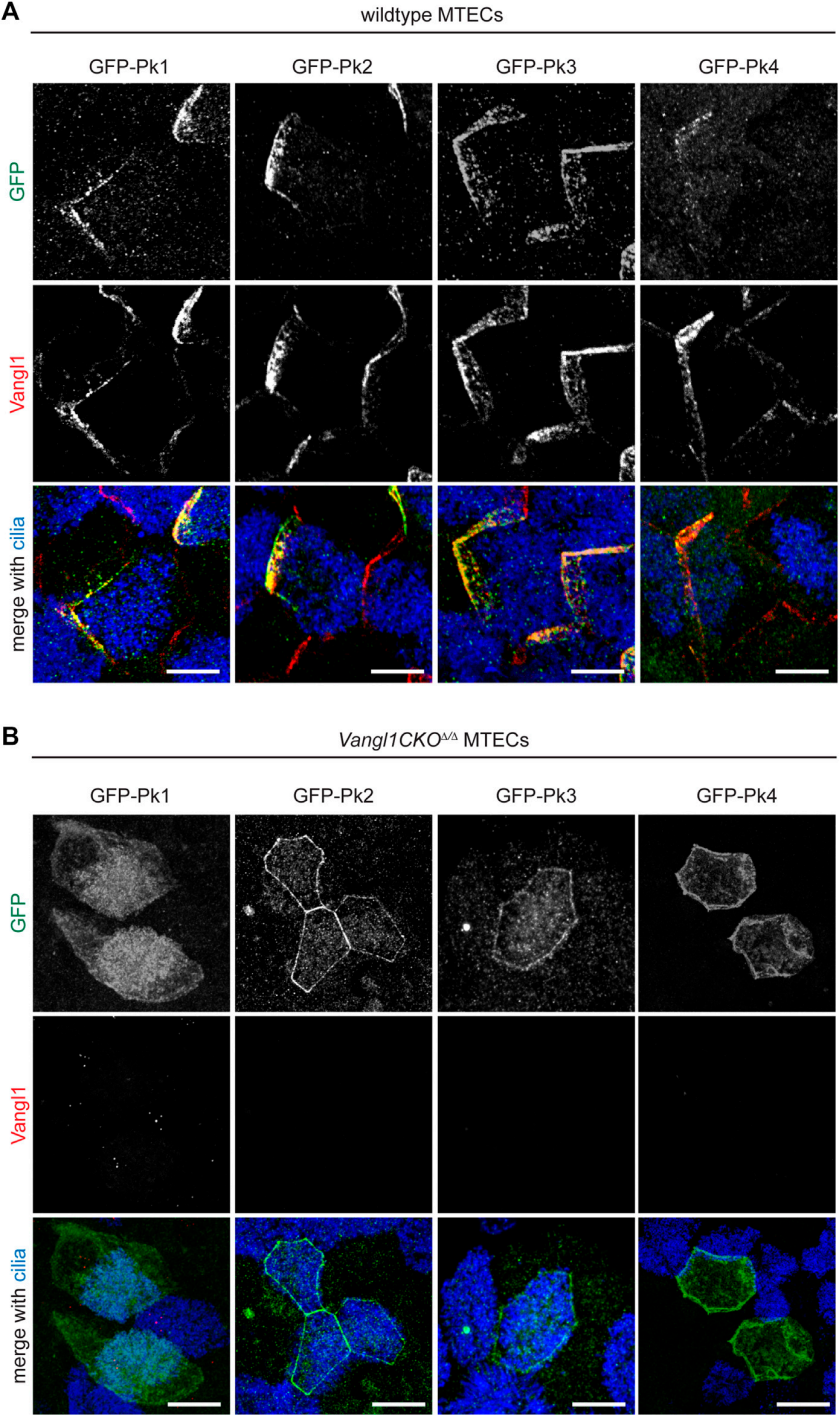
Vangl1 is required for Pk1-4 crescent localization in the airway epithelium. Wildtype **(A)** and *Vangl1CKO* ^*Δ/Δ*^ **(B)** MTECs transduced with GFP-Pk1-4 lentivirus were immunolabeled with GFP (green), Vangl1 (red) and ac. α-Tubulin (blue) antibodies. GFP-Pk1-4 can form crescents in all transduced cells, including both multiciliated and non-ciliated cells in wildtype, but not *Vangl1CKO* ^*Δ/Δ*^ MTECs. Scale bar, 5 μm.

As previously demonstrated using lentiviral expression of GFP-tagged proteins (Vladar et al., 2016), all four Pk proteins can localize asymmetrically in crescents (Figure 2A). Crescents are detected in both multiciliated cells and non-multiciliated cells due to forced expression in all cell types under a ubiquitously active promoter. To determine the dependence of this asymmetric localization on PCP signaling, GFP-Pk proteins were similarly expressed in *Vangl1CKO* ^*Δ/Δ*^ mutant MTECs (Figures 2A,B). In this mutant background, apico-lateral membrane localization of Vangl2 is dramatically reduced and Fz6 and Celsr1 crescents are eliminated (Vladar et al., 2012; Vladar et al., 2016). We observed that the asymmetric localization of all four Pk proteins depends on intact Vangl1 expression. Substantially less Pk1 localizes to apico-lateral membranes, whereas Pk2, Pk3 and Pk4 remain localized to the apico-lateral membrane but do not localize asymmetrically in crescents (Figure 2B).

### Prickle1 and Prickle2 coordinately regulate ciliary polarity

We previously demonstrated that adult *Pk2* ^*−/−*^ mice have only a modest ciliary orientation phenotype (Vladar et al., 2016). We explored whether other Pks might cooperate with Pk2 in establishing or maintaining ciliary polarity in the airway epithelium. Of the other three, only Pk1 mutant mice are available (Tao et al., 2009; Tao et al., 2011). To assess polarity phenotypes, we maintained mice on a C57BL/6 and CBA mixed background. In this background, the *Pk2* ^*−/−*^ strain shows acceptable viability, but we did not obtain *Pk1* ^*−/−*^ homozygotes that survive past early embryonic development (not shown).

**FIGURE 3:**
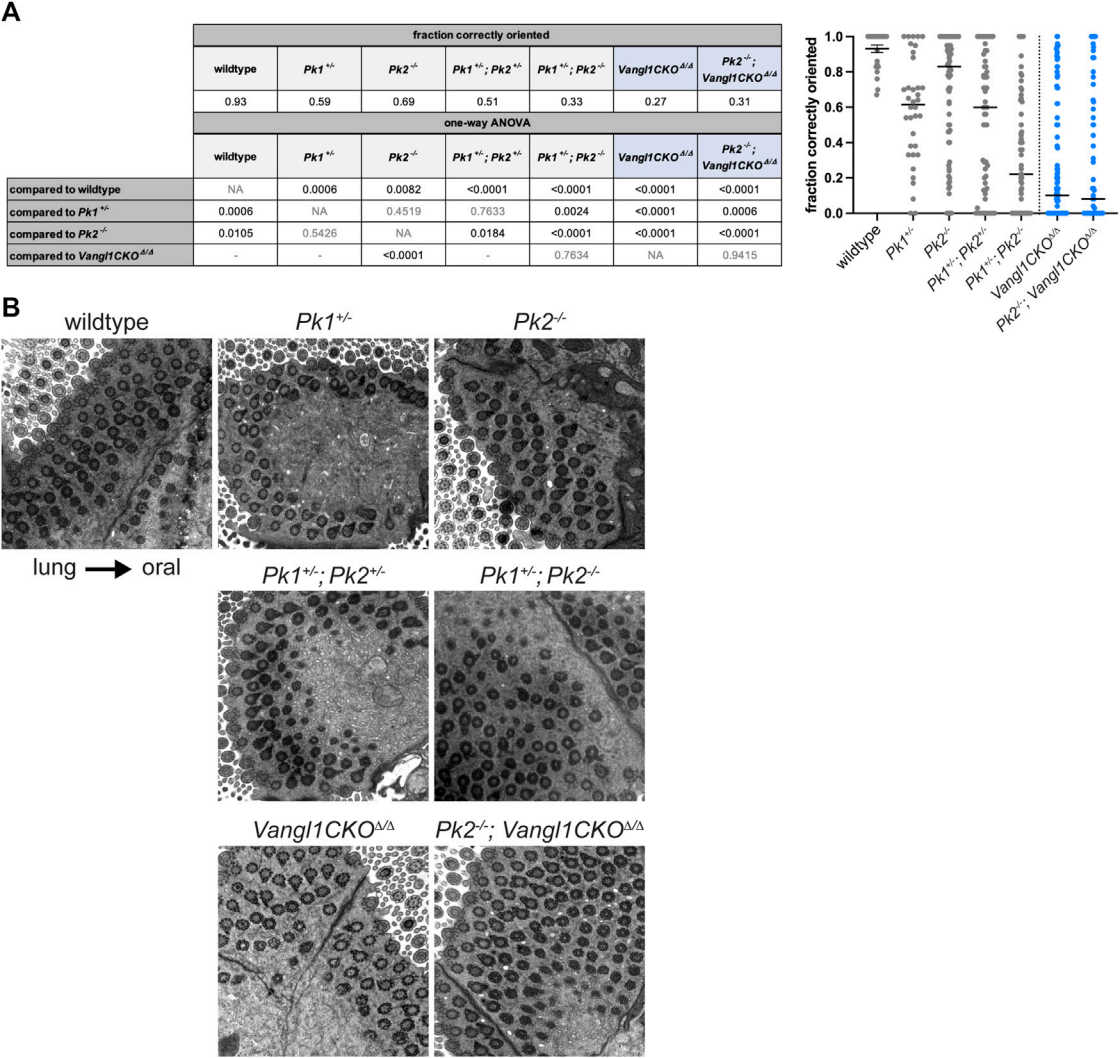
Basal body orientation in adult *Pk* mutant multiciliated cells. **(A)**. Basal body misorientation metrics in adult Pk mutants with appropriate statistics. *Vangl1CKO* ^*Δ/Δ*^ and *Pk2* ^*−/−*^ data were used from (Vladar et al., 2016) with permission. Fraction of correctly oriented basal feet per cell is plotted in the graph (right). Results from a one-way ANOVA are shown in the table (left) as follows: statistically significant *p*-values are shown in black, nonsignificant *p*-values are shown in grey, grey "-” indicates tests that are not shown. *Vangl1CKO* ^*Δ/Δ*^ containing genotypes are highlighted in blue. **(B)**. Representative TEM images showing basal foot orientation with respect to tissue axis.

Polarity phenotypes are assessed in tracheal multiciliated cells using TEM of basal feet, the polarized appendages present on cilia that point towards the direction of the ciliary beat (Boisvieux-Ulrich et al., 1985; Vladar et al., 2015) . Because polarity continues to refine up to postnatal week three (Vladar et al., 2012; Francis and Lo, 2013), age-matched samples were evaluated at approximately 3–6 months of age. We used a scoring system that measures the percentage of anatomically correctly vs. incorrectly oriented basal feet within a tissue sample (Supplementary Figure S3A). By this metric, we show that in wildtype adult mice approximately 93% of basal feet are oriented within ±15° of the oral-lung axis, while in *Pk2* ^*−/−*^ adults only approximately 69% of basal feet are correctly polarized (p-val = 0.0082, Figures 3A,B). *Pk2* ^*+/−*^ heterozygotes are phenotypically normal (not shown). In comparison, *Vangl1CKO* ^*Δ/Δ*^ animals display severely misoriented cilia with only approximately 27% of basal feet are oriented proximally (p-val < 0.0001, Figures 3A,B).

To ask if Pk1 might participate with Pk2 in establishing PCP, we examined adult *Pk1* ^*+/−*^ heterozygotes, *Pk1* ^*+/−*^ *; Pk2* ^*+/−*^ double heterozygotes, and *Pk1* ^*+/−*^ *; Pk2* ^*−/−*^ mutants (Figures 3A,B and Supplementary Figure S3B). *Pk1* ^*+/−*^ heterozygotes also showed a modest polarity phenotype (59% correctly oriented, p-val = 0.0006), which was not statistically different from *Pk2* ^*−/−*^ (p-val = 0.4519). The *Pk1* ^*+/−*^ *; Pk2* ^*+/−*^ (51% correctly oriented, p-val < 0.0001) and *Pk1* ^*+/−*^ *; Pk2* ^*−/−*^ (33% correctly oriented, p-val < 0.0001) double mutants both had misoriented cilia. The phenotypes in both were significantly worse (*Pk1* ^*+/−*^ *; Pk2* ^*+/−*^ p-val = 0.0184; *Pk1* ^*+/−*^ *; Pk2* ^*−/−*^ p-val < 0.0001) compared to *Pk2* ^*−/−*^. The *Pk1* ^*+/−*^ *; Pk2* ^*+/−*^ phenotype was trending towards worse, but was not significant, while the *Pk1* ^*+/−*^ *; Pk2* ^*−/−*^ phenotype was significantly worse (p-val = 0.0024) compared to *Pk1* ^*+/−*^. These data suggest overlapping functions for these isoforms. *Pk2* ^*−/−*^ homozygosity combined with *Vangl1CKO* ^*Δ/Δ*^ did not further enhance the *Vangl1CKO* ^*Δ/Δ*^ phenotype (p-val = 0.9415). The severity of the *Pk1* ^*+/−*^ *; Pk2* ^*+/−*^ double heterozygotes was less (p-val < 0.0001), while that of *Pk1* ^*+/−*^ *; Pk2* ^*−/−*^ was not statistically different (p-val = 0.7634) from that of *Vangl1CKO* ^*Δ/Δ*^ adults.

**FIGURE 4:**
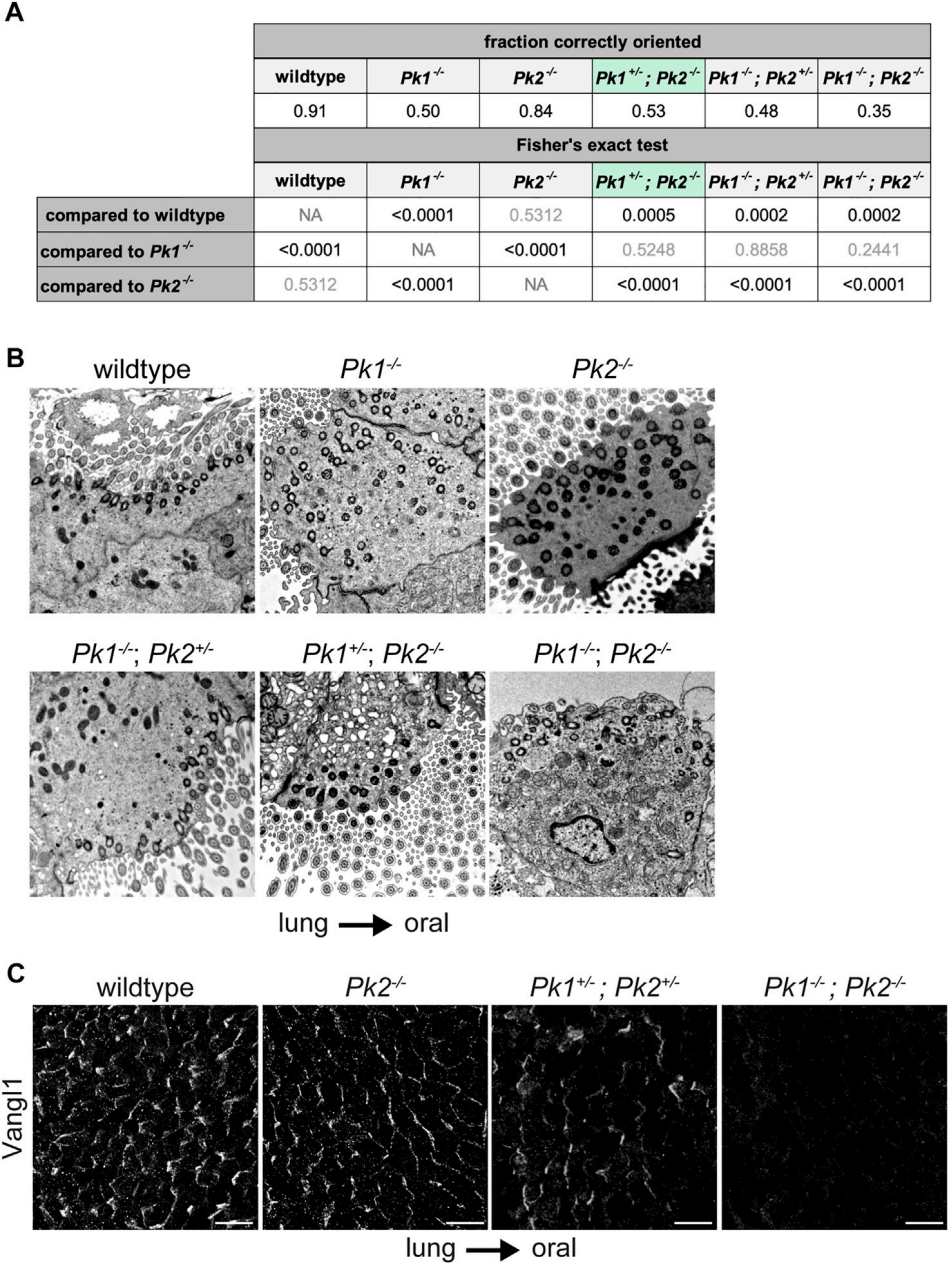
Basal body orientation in perinatal *Pk* mutant multiciliated cells. **(A)**. Basal body misorientation metrics in perinatal Pk mutants with appropriate statistics. Results from a Fisher’s exact test are shown in the table (left) as follows: statistically significant *p*-values are shown in black, nonsignificant *p*-values are shown in grey. *Pk1* ^*+/−*^; *Pk2* ^*−/−*^ is highlighted in green to indicate that mice were analyzed at P16. **(B)**. Representative TEM images showing basal foot orientation with respect to tissue axis. **(C)**. Maximum projections of confocal image stacks of wholemount immunolabeled mouse perinatal tracheal lumens with Vangl1 antibody. Scale bar, 20 μm. Note, due to tissue curvature, it was not always possible to mount the samples sufficiently level to capture the entire tissue layer in a single image stack.

To further analyze Pk1 and Pk2 combinatorial phenotypes, we aimed to obtain *Pk1* ^*−/−*^, *Pk1* ^*−/−*^ *; Pk2* ^*+/−*^, *Pk1* ^*+/−*^ *; Pk2* ^*−/−*^ and *Pk1* ^*−/−*^ *; Pk2* ^*−/−*^ mice. We acquired these genotypes from different Pk1 and Pk2 mutant lines (Minegishi et al., 2017). In a predominantly C57BL/6 background, *Pk1* ^*−/−*^ homozygotes from this strain survive embryonic development but die at postnatal day 0 (P0) (Tao et al., 2012). They display relatively fewer cilia per multiciliated cell, and weak viability, making their polarity phenotype difficult to analyze. Nevertheless, basal body misorientation was readily apparent in TEM images (Figures 4A,B, Supplementary Figure S3C and Supplementary Table S3). We observed that while littermate control animals show >90% of basal feet within ±15° of the oral-lung axis, only approximately 50% of basal feet in *Pk1* ^*−/−*^ homozygotes are correctly polarized (p-val < 0.0001, Figures 4A,B). While *Pk2* ^*−/−*^ polarity was not significantly worse than wildtype control with this allele and genetic background (p-val = 0.5312), additionally removing one copy of *Pk1* (*Pk1* ^*+/−*^ *; Pk2* ^*−/−*^) worsens the phenotype to reach significance relative to both wildtype control (p-val = 0.0005) and *Pk2* ^*−/−*^ (p-val < 0.0001). Note that *Pk1* ^*+/−*^ *; Pk2* ^*−/−*^ were evaluated at P16, so the perinatal phenotype is likely somewhat worse. *Pk1* ^*−/−*^ *; Pk2* ^*−/−*^ double homozygotes show a trend toward a stronger polarity disruption compared to *Pk1* ^*−/−*^, at approximately 35% correctly polarized, though we were able to count relatively few basal bodies and this difference did not reach statistical significance (p-val = 0.2441). Consistent with these data suggesting partially overlapping function, *Pk1* ^+/−^ *; Pk2* ^+/−^ double heterozygotes retain some asymmetric apico-lateral localization of Vangl1 as seen in wildtype animals (Vladar et al., 2012), but the double homozygotes have barely detectible Vangl1 (Figure 4C). From these results, we conclude that Pk1 and Pk2 have partially overlapping function in establishing tracheal epithelial polarity, with Pk1 playing a greater role than Pk2.

Notably, E18.5 pups recovered from a *Pk1* ^+/−^ *; Pk2* ^+/−^ x *Pk1* ^+/−^ *; Pk2* ^+/−^ cross show that *Pk1* ^*−/−*^ homozygotes are smaller than those with a heterozygous Pk1 mutation and display an orofacial defect that may interfere with feeding and thereby cause mortality (Supplementary Figure S4A). The *Pk1* ^*−/−*^ *; Pk2* ^*−/−*^ double homozygotes, in addition to the orofacial phenotype, are yet smaller than the *Pk1* ^*−/−*^ homozygotes and two of ten double homozygotes displayed neural tube defects including craniorachischisis and spina bifida (Supplementary Figure. S4B). Also of note, approximately 70% of *Pk1* ^*+/−*^ *; Pk2* ^*−/−*^ mutant mice die before weaning with severe growth retardation (not shown). Pk1 and Pk2 therefore also appear to have overlapping functions in developmental processes other than airway epithelial polarization.

## Discussion

Here, we demonstrate that Pk1 and Pk2 play quantitatively and qualitatively distinct roles in the planar polarization of the airway epithelium. The *Pk1* ^*−/−*^ mutant phenotype is more severe than that of *Pk2* ^*−/−*^, but genetic interaction between the heterozygotes indicates that they both contribute, directly or indirectly, to a common process. Based on our data, we cannot rule out that the remaining Pk isoforms might additionally contribute to planar polarization.

The distinct spatial and temporal expression patterns of Pk1 and Pk2 imply differing roles. We suggest that the expression of Pk1 in all cells of the immature epithelium is indicative of a critical role in establishing initial polarization, and that its initial high and then declining expression indicates that after polarity establishment its role is diminished. Pk3 is also expressed in all cells at a modestly increasing level with maturation. Pk1 and Pk3 are the two Pk isoforms expressed uniformly in all cells of the epithelium. Since we expect that tissue-wide signaling is required to maintain PCP, we hypothesize that Pk3 assumes a function in a maturing epithelium that is originally fulfilled by Pk1 in the immature epithelium. We note, however, that if the *Pk3* mRNA detected in immature cultures is translated, and if that protein assumes an apico-lateral localization, it is unable to fully compensate for the loss of Pk1. Pk1 may have a unique activity required during initial polarization that is not required for polarity maintenance. In *Drosophila*, Pk isoforms have been shown to compete for participation in PCP core signaling (Ayukawa et al., 2014; Ambegaonkar and Irvine, 2015; Cho et al., 2020; Cho et al., 2022), so perhaps Pk3 protein is only able to enter PCP signaling complexes when declining Pk1 levels allow for its incorporation. While the dynamics and contribution of Pk3 to PCP signaling remain to be explored, the expression of Pk1 in all cells, at least in the immature epithelium, is consistent with the prevailing notion that a full complement of core PCP proteins is required for the local coordination of polarity among cells in the tissue.

Unlike Pk1 and Pk3, Pk2 and Pk4 expression are limited to multiciliated cells, suggesting that these isoforms are responsible for a specific polarization function in these cells. Orientation of basal feet in multiciliated cells is known to depend on their assembly into a network of actin and microtubules, and this structure must also be oriented according to the polarization axis of the tissue (Mitchell et al., 2007; Vladar et al., 2012). The phenotype of Pk2 mutants suggests Pk2 is likely to participate in both processes. The similar exclusive expression of Pk4 in multiciliated cells suggests the possibility that Pk4 may also participate in these processes. Partially redundant function for core PCP protein isoforms is not a unique idea; for example, in the closing neural tube (Torban et al., 2008; Antic et al., 2010; Song et al., 2010), and in the airway epithelium (Vladar et al., 2012), Vangl1 and Vangl2 have been shown to have distinct but overlapping functions though the precise nature of the overlap and differences is not known.

## Conclusion

Through the use of mutant mouse models, we began deciphering the complex, but critical contribution of Pk family core PCP proteins to the regulation of airway epithelial planar polarity. Eventual development of Pk3 and Pk4 knockout models will lead to broader insight into specific roles of distinct paralogs.

## Data Availability

The raw data supporting the conclusions of this article will be made available by the authors, without undue reservation.

## Abbreviations

PCP, planar cell polarity; Pk, Prickle; MTEC, mouse tracheal epithelial cell; ALI, air-liquid interface; TEM, transmission electron microscopy.
