# Supplementary material for: Distinct overlapping functions for Prickle1 and Prickle2 in the polarization of the airway epithelium
Source: Front Cell Dev Biol. 2022 Sep 13;10:976182. doi: 10.3389/fcell.2022.976182 (PMC9513604; doi:10.3389/fcell.2022.976182)
Supplement: Supplementary file 2 [file DataSheet3.pdf]

Supplemental Materials

Inventory:

1. 4 Supplemental Figures
2. 3 Supplemental Tables

Supplemental Figures

Figure S1. **Alignment of mouse Pk1-4 protein sequences.** PET (green) and LIM (red) domains are highlighted. The four C-terminal amino acids represent the CAAX motif.

```
MmPk4      ----------------------------------MSVQNSDWSL---------------- 10
MmPk3      MFARGSRRRRSGRAPPEAEDPARGQPCNSCREQCPGFLLHGWRKICQHCKCPREEHAVRT 60
MmPk1      ------------------------------------------------------------ 0
MmPk2      --------------------------------------------------------MVTV 4

MmPk4      -------QQDNPIFREPDPPVYTDSDSGRRPVEDYEDTSAQAATCSSLGPPCLD------ 57
MmPk3      VPVDLERIMCRLISDF-QRHSISDDDSGC-ASEEY-----------AWVPPGLKPEQVYQ 107
MmPk1      MPLEMEPKMSKLVFGC-QRSSTSDDDSGC-ALEEY-----------AWVPPGLRPEQIQL 47
MmPk2      MPLEMEKTISKLMFDF-QRSSTSDDDSGC-ALEEY-----------AWVPPGLKPEQVHQ 51
                     . :    :    :*.***    *:*           :  ** *

MmPk4      ------------INQVSNWPGFRTLLQQLPPQDSDERYCLALGEEELAQLRLFCAQRKQK 105
MmPk3      FFSCLPEDKVPYVNSPGEKYRIKQLLHQLPPHDSEAQYCTALEEEEKKELRAFSQQRKRE 167
MmPk1      YFACLPEEKVPYVNSPGEKHRIKQLLYQLPPHDNEVRYCQSLSEEEKKELQVFSAQRKKE 107
MmPk2      YYSCLPEEKVPYVNSAGEKLRIKQLLHQLPPHDNEVRYCNSLDEEEKRELKLFSNQRKRE 111
                       :*. .:   :: ** ****:*.: :** :* ***  :*: *. ***::

MmPk4      SLGQGVARLLPPKLEGYTCKKCKKLLDPGEYGVFAARAGEQSCWHRPCFACQACGQGLIN 165
MmPk3      NLGRATVRIFPVTITGAICEECGKQIGGGDIAVFASRAGLGACWHPQCFVCTTCQELLVD 227
MmPk1      ALGRGTIKLLSRAVMHAVCEQCGLQMNGGEVAVFASRAGPGVCWHPSCFVCFTCNELLVD 167
MmPk2      NLGRGNVRPFPVTMTGAICEQCGGQIKGGDIAVFASRAGHGICWHPPCFVCTVCNELLVD 171
            **:.  : :   :    *::*   :  *: .***:***   ***  **.* .* : *::

MmPk4      LIYFYHEGHLYCGRHHAELLRPRCPACDQLIFSQRCTEAEGQRWHENHFCCQDCAGPLDG 225
MmPk3      LIYFYHAGKVYCGRHHAECLRPRCQACDEIIFSPECTEAEGRHWHMGHFCCFECEASLGG 287
MmPk1      LIYFYQDGKIHCGRHHAELLKPRCSACDEIIFADECTEAEGRHWHMKHFCCLECETVLGG 227
MmPk2      LIYFYQDGKIYCGRHHAECLKPRCAACDEIIFADECTEAEGRHWHMRHFCCFECETVLGG 231
           *****: *:::******* *:*** ***::**: .******::**  **** :*   *.*

MmPk4      GRYALPGGSPCCPSCFSKRYRSA---GSSSVGVAEGQASFEEEGPDPSVGWNRASSDDKI 282
MmPk3      QRYVMRQSRPHCCACYEARHAEYCDGCGEHIGLDQGQMAYEGQH------WHASDRCFCC 341
MmPk1      QRYIMKDGRPFCCGCFESLYAEYCETCGEHIGVDHAQMTYDGQH------WHATEACFSC 281
MmPk2      QRYIMKEGRPYCCHCFESLYAEYCDTCAQHIGIDQGQMTYDGQH------WHATETCFCC 285
            ** :  . * *  *:.  : .     .. :*: ..* ::: :       *: :.

MmPk4      TSRAALLSAVPTLTLETLN----GASKRQDRDGPQTPGSPIEDS---------------- 322
MmPk3      SRCSRPLLGRPFLPRRGLIFCSRACSLGSETTAPGPGRRSWSAGTVTTPLTTSTASFSAT 401
MmPk1      AQCKASLLGCPFLPKQGQIYCSKTCSLGEDIHASDSSDSAFQSARSRDSRRS--VRMGRS 339
MmPk2      AHCKKSLLGRPFLPKQGQIFCSRACSAGEDPNGSDSSDSAFQNARAKESRRS--AKIGKN 343
           :     * . * *  .        .*  .:  .        . .

MmPk4      ----------------P-------CP---------------TCSSSSE----SEPEGFFF 340
MmPk3      EGTSETASKGTCTKAEPAAGPEEPSHFLRG---APHRHSMPELGLRSAPEPPT------- 451
MmPk1      SRSADQCRQSLLL--SPALNYK--FPGLSGNADDTLSRKLDDVSLASRQGAGFANEEFWK 395
MmPk2      KGKTEEAMLNQ----HSQLQVS--SNRLSA-DVDPLSVQMDLLSLS-SQTPSLNRDPIWR 395
                                                      .

MmPk4      G------QRLPEPWKTPENLQADDRD-I--SR---KHCTIC------------------- 369
MmPk3      -----ESPGHPAPHPDDNAFGRQSTPRVSFRDPLVSEGGPRRTLSAPPAQRRRPRSPPP- 505
MmPk1      ARVEQEASEDPEEWAEHEDY----------MTQLLLKFGDKNLFQ-QQSSEVDPRASEHW 444
MmPk2      SREEPFHYGNKMEQNQSQ-S----------PLQLLSQCNIRTSYS-PGG-QGAGAQPDMW 442
                            :                  .

MmPk4      ------------------------------------------------------------ 369
MmPk3      --------RT----PSCHHH---------------------------------------- 513
MmPk1      IPDNMVTNKP--EVKPNHQGLASKKYQSDMYW-----AQSQDGLGDSAYGSHPGPASSRR 497
MmPk2      AKHFSNPKRSSSMALKGHGGSFIQECREDYYPGRLMSQESYSDMSSQSFNETRGSIPVPK 502

MmPk4      ------------------------------------------------------------ 369
MmPk3      ------------------------------------------------------------ 513
MmPk1      LQEL-DLDHGAAGYTHDQSQWYEDSLECLSDLK-PEQSIRDSMDSLALSNITGASVDGES 555
MmPk2      YEEEEEEEEGGISTQQCRPRRPLSSLKYTEDMTPTEQTPRGSMESLALSNATGLSAEGGA 562

MmPk4      ------------------------------------------------------------ 369
MmPk3      ------------------------------------------------------------ 513
MmPk1      KPRPSLYS--LQNF-EEIEAEDCEKMSNMGTLNSSMLHRSAESLQSLNSGLCPEKILPEE 612
MmPk2      KRQEHLSRFSMPDLSKDSGMNVSEKLSNMGTLNSSMQFRSAESVRSLLSAQQYQEMEGNL 622

MmPk4      ------------------------------------------------------------ 369
MmPk3      --------------------------------------------HHHRRRRQRHR-RRGS 528
MmPk1      KPAHLPVLRR-SKSQSR-PQQVKFSDDVIDNG-SYDIEIRQPPMSERTRRRAYHFEERGS 669
MmPk2      HQLSNPLGYRDLQSHGRMHQSFDFDGGIASSKLPGQEGVHIQPMSERTRRRTTSRD-DNR 681

MmPk4      ------------------------------------------------------------ 369
MmPk3      HHHHHHPGRHGHHRC--------------------------------------------- 543
MmPk1      RPHHHRHRRSRKSRSDNALNLVTERKYSA--KDRLRLYTPDNYEKFIQNKSARELQAYMQ 727
MmPk2      RFRPHRSRRSRRSRSDNALHLASEREVIARLKERPPLRAREDYDQFMRQRSFQESLGQGS 741

MmPk4      ------------------------------------------------------------ 369
MmPk3      -------------D-------------LGSGSDSGSCSSSPSSPSSESSEDDGFFLGERI 577
MmPk1      NANLYSQYAHATSDYALQNPGMNRFLGLCGEDDDSWCS---SSTSSSDSEEEGYFLGQPI 784
MmPk2      RRDLYSQCPRTVSDLALQNAFGERWGPYF--TEYDWCS---TCSSSSESDNEGYFLGEPI 796

MmPk4      -------------------------------------------------    369
MmPk3      PLPPHLCRPRTTQDTSTET-FNSPAQPLVQE-SHPVMPRQTRDKNCIVA    624
MmPk1      PQPRPQRFTYYTDDLSSPA-SALPTPQFTQRTTKSKKKKGHKGKNCIIS    832
MmPk2      PQPARLRYVTSDELLHKYSSYGVPKSSTLGGRGQLHSRKRQKSKNCIIS    845
```

Figure S2. **Schematics of MTEC culture ciliogenesis and *Pk* expression. A.** Schematic of ciliogenesis during MTEC culture progression. Ciliated cell formation begins at ALI+2d. PCP acquisition indicated by crescent formation is evident slightly prior to the appearance of cilia. **B.** Schematic of *Pk1-4* expression during MTEC timecourse. **C.** Schematic of *Pk1-4* cell type specific enrichment in the airway epithelium.

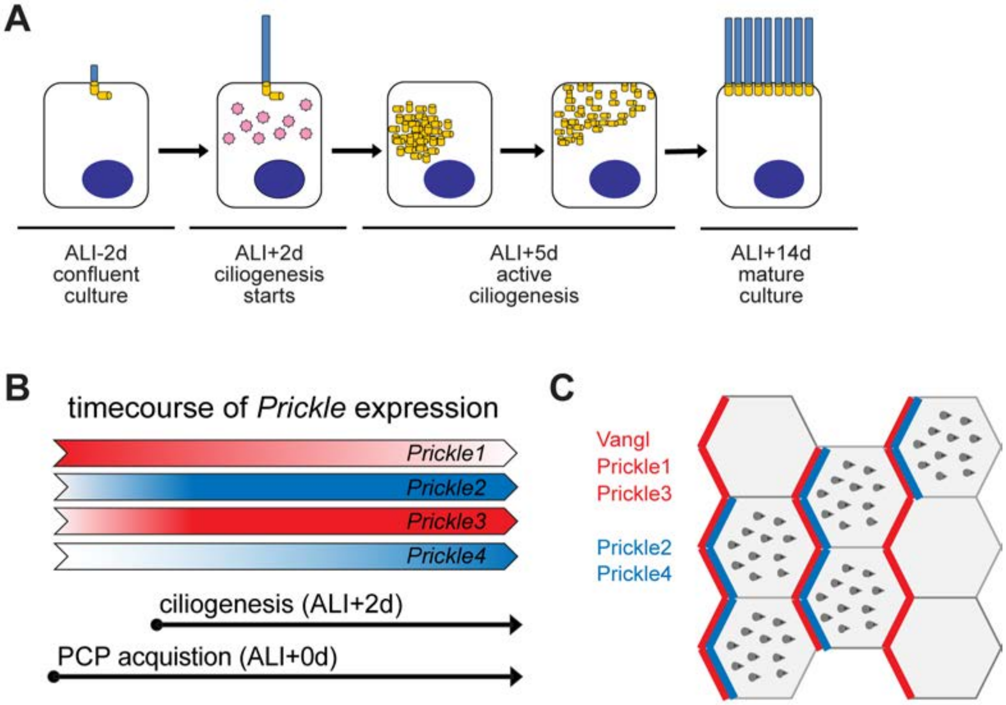

Figure S3. **Basal body misorientation in Pk mutants. A.** Example TEM image of basal feet on cilia and determination of ciliary orientation. Arrows point to basal feet. **B-C.** Table summary of the number of basal feet quantitated in adult (**B**) and perinatal (**C**) mice.

A

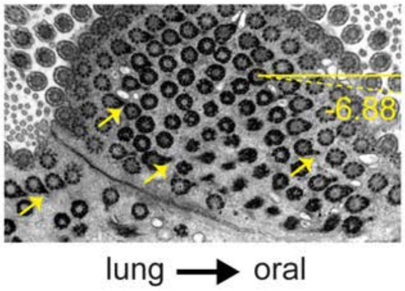

B

	genotypes						
	wildtype	*Pk1* $^{+/-}$	*Pk2* $^{-/-}$	*Pk1* $^{+/-}$; *Pk2* $^{+/-}$	*Pk1* $^{+/-}$; *Pk2* $^{-/-}$	*Vangl1CKO* $^{\Delta/\Delta}$	*Pk2* $^{-/-}$; *Vangl1CKO* $^{\Delta/\Delta}$
cells analyzed (n)	28	36	77	63	63	88	70
basal bodies analyzed (n)	277	444	1,039	743	595	1220	1060
ave. basal bodies per cell +/- SE	10.65 +/- 1.4	12.33 +/- 1.03	13.49 +/- 0.79	11.79 +/- 1.26	9.44 +/- 0.39	13.86 +/- 0.78	15.14 +/- 1.16

C

	genotypes					
	wildtype	*Pk1* $^{-/-}$	*Pk2* $^{-/-}$	*Pk1* $^{+/-}$; *Pk2* $^{-/-}$	*Pk1* $^{-/-}$; *Pk2* $^{+/-}$	*Pk1* $^{-/-}$; *Pk2* $^{-/-}$
mouse age	E18.5	P0	P0	P16	P0	E18.5
mice (n)	3	2	2	1	2	3
basal feet analyzed (n)	23	175	133	180	67	20
correctly oriented (n)	21	87	112	96	32	7
incorrectly oriented (n)	2	88	21	84	35	13

Figure S4. **Developmental phenotypes in Pk mutants. A.** Craniofacial and **B.** neural tube closure defects in Pk mutant litter mates from a $Pk1^{+/-}$; $Pk2^{+/-}$ x $Pk1^{+/-}$; $Pk2^{+/-}$ cross.

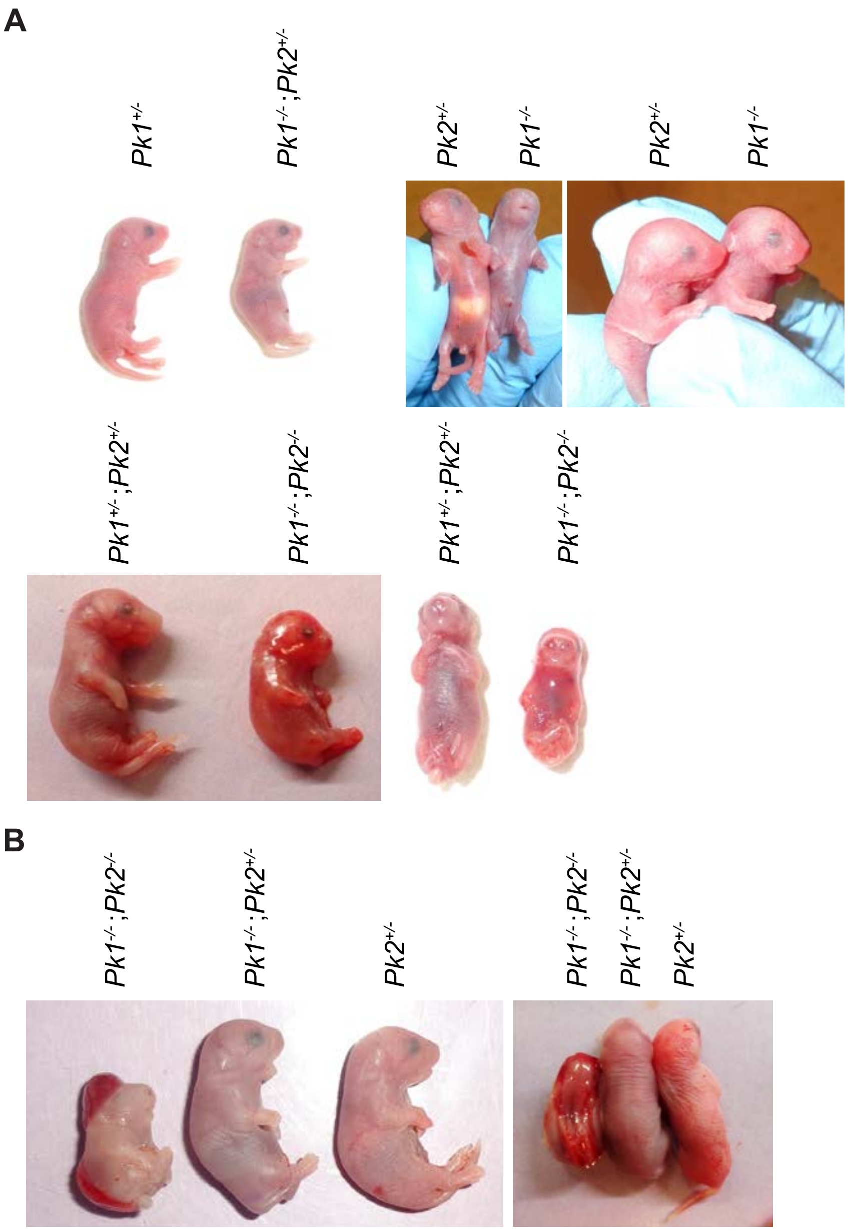

Supplemental Tables

Table S1. **Antibodies.**

Protein	Antibody	Source
**Vangl1**	HPA025235	Sigma Aldrich
**Prickle1**	-	Gibbs *et al*., 2016
**Prickle2**	-	Deans *et al*., 2007
**Frizzled6**	AF1526	R&D Systems
**ac. α-Tubulin**	ab24610	Abcam
**GFP**	4745-1051	AbD Serotech

Table S2. **Primer sequences.**

Target	Forward primer sequence	Reverse primer sequence
MmPrickle1	GATGGAGAAAGCAAGCCAAG	TGTGCAGCATGGAAGAGTTC
MmPrickle2	ACATGGGCACTCTCAACTCC	TGTATCCTAGGGGGTTGCTG
MmPrickle3	TGCTGTTTCGAGTGTGAAGC	CATCACAGTATTCCGCATGG
MmPrickle4	CCACAGGACAGTGATGAACG	CCTTCAAGCTTAGGAGGCAG
MmCentrin2	ACAGGGCAGAACAAGAGCAC	CCACTGCTTATGGTGACATGG
MmGapdh	GACTTCAACAGCAACTCCCAC	TCCACCACCCTGTTGCTGTA

Table S3. **Basal feet quantitation of Pk mutant mice from Fig. 4.**

wildtype				*Pk1*$^{-/-}$				*Pk2*$^{-/-}$				*Pk1*$^{+/-}$; *Pk2*$^{-/-}$				*Pk1*$^{-/-}$; *Pk2*$^{+/-}$				*Pk1*$^{-/-}$; *Pk2*$^{-/-}$				
cell	BB - correct	BB - incorrect	**BB - total**	cell	BB - correct	BB - incorrect	**BB - total**	cell	BB - correct	BB - incorrect	**BB - total**	cell	BB - correct	BB - incorrect	**BB - total**	cell	BB - correct	BB - incorrect	**BB - total**	cell	BB - correct	BB - incorrect	**BB - total**	
1	4	1	**5**	1	1	3	**4**	1	5	0	**5**	1	4	1	**5**	1	2	2	**4**	1	1	0	**1**	
2	3	0	**3**	2	2	5	**7**	2	8	0	**8**	2	8	11	**19**	2	0	2	**2**	2	0	1	**1**	
3	3	0	**3**	3	1	5	**6**	3	3	4	**7**	3	0	5	**5**	3	2	3	**5**	3	2	2	**4**	
4	1	0	**1**	4	3	3	**6**	4	10	1	**11**	4	11	5	**16**	4	0	2	**2**	4	2	0	**2**	
5	1	0	**1**	5	4	1	**5**	5	8	1	**9**	5	21	0	**21**	5	2	0	**2**	5	0	1	**1**	
6	4	0	**4**	6	3	7	**10**	6	5	0	**5**	6	2	10	**12**	6	1	3	**4**	6	1	0	**1**	
7	1	0	**1**	7	2	1	**3**	7	7	0	**7**	7	18	18	**36**	7	2	1	**3**	7	0	1	**1**	
8	1	1	**2**	8	5	0	**5**	8	4	4	**8**	8	5	7	**12**	8	2	3	**5**	8	0	1	**1**	
9	1	0	**1**	9	3	0	**3**	9	13	1	**14**	9	5	1	**6**	9	1	1	**2**	9	0	1	**1**	
10	2	0	**2**	10	4	4	**8**	10	4	3	**7**	10	4	3	**7**	10	3	3	**6**	10	0	3	**3**	
				11	0	3	**3**	11	8	0	**8**	11	3	1	**4**	11	4	1	**5**	11	0	1	**1**	
				12	0	2	**2**	12	8	0	**8**	12	2	2	**4**	12	1	4	**5**	12	0	1	**1**	
				13	2	0	**2**	13	8	2	**10**	13	1	1	**2**	13	4	2	**6**	13	0	2	**2**	
				14	3	10	**13**	14	4	0	**4**	14	3	2	**5**	14	1	5	**6**	14	1	0	**1**	
				15	1	0	**1**	15	5	1	**6**	15	2	2	**4**	15	3	0	**3**					
				16	3	6	**9**	16	4	1	**5**	16	0	1	**1**	17	4	3	**7**					
				17	2	1	**3**	17	5	2	**7**	17	1	2	**3**									
				18	0	8	**8**	18	3	1	**4**	18	5	3	**8**									
				19	5	0	**5**					19	0	6	**6**									
				20	12	0	**12**					20	1	3	**4**									
				21	2	3	**5**																	
				22	4	0	**4**																	
				23	3	3	**6**																	
				24	3	2	**5**																	
				25	2	2	**4**																	
				26	3	3	**6**																	
				27	4	2	**6**																	
				28	0	3	**3**																	
				29	4	3	**7**																	
				30	0	3	**3**																	
				31	0	3	**3**																	
				32	4	0	**4**																	
				33	2	2	**4**																	
	**21**	**2**	**23**		**87**	**88**	**175**		**112**	**21**	**133**		**96**	**84**	**180**		**32**	**35**	**67**		**7**	**14**	**21**	**TOTAL (n)**
